# Clinical profile of comorbidity of rare diseases in a Tunisian patient: a case report associating incontinentia pigmenti and Noonan syndrome

**DOI:** 10.1186/s12887-018-1259-8

**Published:** 2018-08-29

**Authors:** Nehla Ghedira, Arnaud Lagarde, Karim Ben Ameur, Sahar Elouej, Rania Sakka, Emna Kerkeni, Fatma-Zohra Chioukh, Sylviane Olschwang, Jean-Pierre Desvignes, Sonia Abdelhak, Valerie Delague, Nicolas Lévy, Kamel Monastiri, Annachiara De Sandre-Giovannoli

**Affiliations:** 1 Research Unit 01/UR/08-14, Faculty of Medicine of Monastir, University of Monastir, Avenue Avicenne, 5019 Monastir, Tunisia; 2 Aix Marseille University, INSERM, GMGF, Marseille, France; 3 Department of Intensive Care and Neonatal Medicine, Fattouma Bourguiba University Hospital, Monastir, Tunisia; 4 Departement of Medical Genetics, Childrens’ Hospital La Timone, 264 Rue Saint Pierre, Marseille, France; 5 Groupe Ramsay Générale de Santé, Hôpital Clairval, Marseille, France; 6 Institut Pasteur de Tunis, Laboratoire de Genomique Biomedicale et Oncogenetique LR11IPT05, Tunis, Tunisia

**Keywords:** Noonan syndrome, RAS-MAPK pathway, RAF1, Dysmorphism, Incontinentia Pigmenti, X-linked disorder, Comorbidity, Next generation sequencing

## Abstract

**Background:**

Noonan syndrome (NS) is an autosomal dominant multisystem disorder caused by the dysregulation of several genes belonging to the RAS Mitogen Activated Protein Kinase (MAPK) signaling pathway. Incontinentia Pigmenti (IP) is an X-linked, dominantly inherited multisystem disorder.

**Case presentation:**

This study is the first report of the coexistence of Noonan (NS) and Incontinentia Pigmenti (IP) syndromes in the same patient. We report on the clinical phenotype and molecular characterization of this patient. The patient was examined by a pluridisciplinary staff of clinicians and geneticist. The clinical diagnosis of NS and IP was confirmed by molecular investigations. The newborn girl came to our clinics due to flagrant dysmorphia and dermatological manifestations. The clinical observations led to characterize the Incontinentia Pigmenti traits and a suspicion of a Noonan syndrome association. Molecular diagnosis was performed by Haloplex resequencing of 29 genes associated with RASopathies and confirmed the NS diagnosis. The common recurrent intragenic deletion mutation in *IKBKG* gene causing the IP was detected with an improved PCR protocol.

**Conclusion:**

This is the first report in the literature of comorbidity of NS and IP, two rare multisystem syndromes.

## Background

Noonan syndrome (NS, OMIM 163950) is one of the most frequent genetic disorders in children with an autosomal dominant pattern of inheritance [1, 2]. This multisystem disease is variably expressed with an estimated prevalence of 1 in 1000–2500 live births [1, 3].

The clinical diagnosis of NS is established on the basis of distinctive features according to different criteria developed by Van Der Burgt and *al* [4] in 1994. Constant clinical traits characterizing this syndrome include: dysmorphic facial features, variable developmental delay with constant short stature, congenital heart defects and chest wall anomaly often consisting of pectus carinatum or/and pectus excavatum [5–7]. NS has also a distinct constellation of ectodermal manifestations that depends on the mutated gene including short and curly hair, loose anagen hair, absent eye-brows, erythema, and granular cell tumors, abnormal pigmented lesions (multiple pigmented naevi, “café au lait” spots and freckles. Keratosis pilaris can also occurs [8–11].

NS has a heterogeneous genetic background that involves genes encoding proteins with roles in the RAS MAPK pathway [5, 12–14]. Gain of function mutations in the *PTPN11* gene (OMIM 176876) are found in 40–50% of NS patients [10, 15, 16]. Other mutations in several genes of the cascade are less frequent: e.g. in *SOS1* (OMIM 182530, about 11% of cases), *RAF1* (OMIM 164760, about 5% of cases), *SHOC2* (OMIM 602775, about 2% of cases), *KRAS* (OMIM 190070, about 1.5% of cases), *NRAS* (OMIM 164790, about 0.2% of cases), *CBL* (OMIM 165360), *RIT1* (OMIM 609591) and recently, with the emergence of NGS technologies in the molecular characterization of rare diseases, other genes have been implicated such as *RRAS* (OMIM 165090), *RASA2* (OMIM 601589), *SOS2* (OMIM 601247) and *LZTR1* (OMIM 600774) [5, 17–19]. Nevertheless, about 30% of NS cases remain without molecular confirmation.

Familial Incontinentia Pigmenti (IP, OMIM 308300) is a rare X-linked dominant genodermatosis that affects ectodermal tissues and is usually prenatally lethal in males [20, 21]. However, some affected males have been reported, either presenting with X-chromosome somatic mosaicism or a concomitant diagnosis of Klinefelter syndrome [22]. In total, 900 to 1200 individuals with IP have been reported in the literature.

More than 80% of IP patients have a recurrent genomic large-scale deletion of exons 4–10 of the *NEMO* gene (*IKBKG*, OMIM 300248), which encodes a regulatory component of the IKB Kinase complex, leading to loss of NF-KB activation and increased apoptosis. Although hypomorphic mutations and CNVs have been reported, the architecture of the NEMO/IKBKG locus seems to facilitate genome instability generating the high frequency of de novo rearrangements observed in IP patients [20, 23, 24].

## Case presentation

**Fig. 1:**
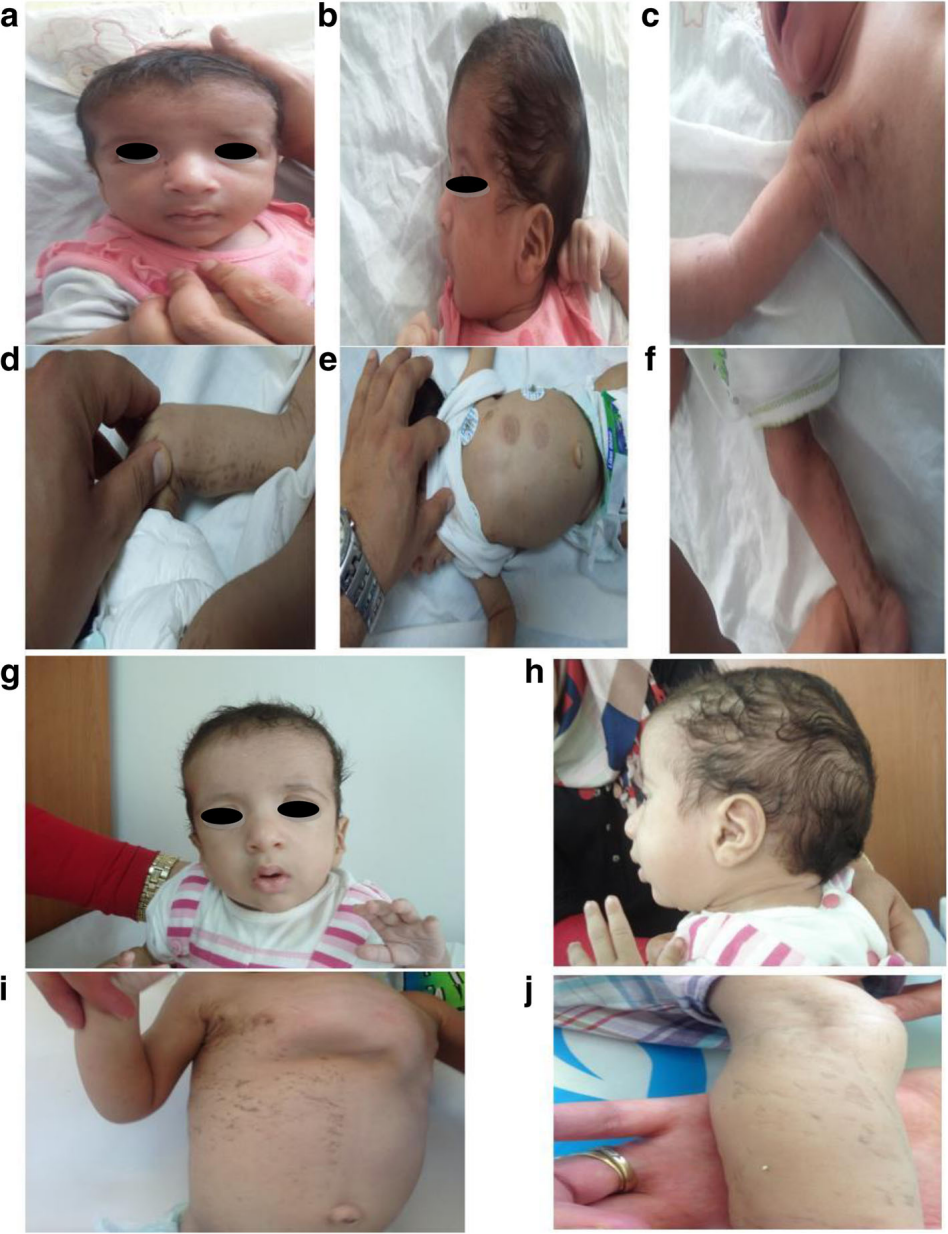
Photographs of the patient showing NS and IP manifestations. **a, b** front and profile photo respectively pointing facial dysmophism at the age of 1 month with broad forehead, hypertelorism, down-slanting palpebral fissures, a high arched palate, bifid uvula and low set posteriorly rotated ears, long philtrum and epicanthal folds; **c-f** typical IP manifestations at the age of 1 month showing linear brownish pigmentation involving the trunk and the extremities; **g, h** front and profile photo of the patient at the age of 1 year highlights the evolution of NS phenotype; **i, j** evolution of the IP manifestations at the age of 1 year, pectus excatavum becomes more pronounced

The patient was a one- month- old baby girl referred to our center for a congenital heart defect. The baby was born at full term (40-week s gestation). Pregnancy, as well as the perinatal period, were unremarkable. The baby had facial dysmorphism with broad forehead, hypertelorism, down-slanting palpebral fissures, a high arched palate, bifid uvula and low set posteriorly rotated ears, long philtrum and epicanthal folds (Fig. 1a, b). Echocardiography showed pulmonic stenosis. During the first week of life, the baby developed Incontinentia Pigmenti, a typical skin rush that started with papules, vesicles and pustules on an erythematous base, followed by plaques and warty papules linearly arranged over erythematous lesions, then a linear brownish pigmentation involving the trunk and the extremities developed by the age of 1 month. All these skin lesions were distributed along the lines of Blaschko (Fig. 1c-f). No dental or eye anomalies were observed. She had congenital stridor, delayed neurological development and growth retardation (weight was 4000 g at 2 months, < P3). These findings were consistent with Noonan syndrome associated with Incontinentia Pigmenti based on “Incontinentia Pigmenti diagnostic criteria update” by Minicand *al* [25]. The phenotypes of the two syndromes evolved with the age of the patient (Fig. 1g-j).

## Methods

The clinical data were compiled by clinical geneticists and pediatricians (R. S, K.BA, K.M, and FZ.C). The patient’s parents signed written informed consent to consultation and molecular studies for both diagnostic and research purposes and also for publication of the patient’s pictures. Along this, parents give also their consent for participation in genetic testing in addition to their child. This work complies with the declaration of Helsinki (https://www.wma.net/policies-post/wma-declaration-of-helsinki-ethical-principles-for-medical-research-involving-human-subjects/) and with the ethical guidelines of the institutions involved. Genomic DNA of the patient and her parents was manually extracted from peripheral blood collected in EDTA tubes according to standard salting out methods and purified by QIAmp CDN kit (Qiagen). DNA quality and quantity were measured on Nanodrop Spectrophotometer (Thermo Scientific) and by Qubit ds S Assay on Qubit 2.0 Fluorometer (Thermo Fisher Scientific).

### NGS analysis

A panel of 29 genes was designed using the online Sure Design software (Agilent Technologies). A predesigned Haloplex Noonan panel containing the genes *PTPN11, SOS1, RAF1, KRAS, BRAF, NRAS, HRAS, MAP2K1, MAP2K2, SHOC2, CBL, SPRED1* and *NF1* was used, to which were added other genes involved in RASopathies (the list of all analyzed genes can be provided on request). The size of the final target region was 232.387kpb with 570 amplicons and the mean sequence coverage was 93.73 at 20× priori 99.41% coverage of the target region. The preparation of the libraries was performed using the Haloplex target enrichment system dedicated to Ion Torrent PGM. Massively parallel sequencing was performed on an Ion Torrent PGM (Thermo Fisher Scientific).

Raw data generated by the PGM sequencer were processed by Torrent Suite Software v.4 and aligned using TMAPv.3. Sequence variants were identified using the Variant Caller tool from the Ion Torrent package using default “germline low stringency” parameters (min_cov_each_strand: 0, min_variant_score: 10, min_allele_freq: 0.1, snp_min_coverage: 6 snp and indel; strand_bias: 0.98 snp and 0.85 indel) then prioritized using the in-house software Varaft (http://varaft.eu) that includes Annovar [26] and UMP-Predictor [27] . Variants not previously reported in healthy controls and classified as pathogenic were evaluated for sequencing depth and visually inspected using the Integrative Genomic Viewer (IGV) before validation by Sanger Sequencing using the manufacturer’s protocol for BigDye ® Terminator sequencing kits. Segregation analysis was performed in both parents by Sanger sequencing.

Genes were named following the Hugo gene nomenclature committee guidelines (https://www.genenames.org/); DNA mutations and predicted protein changes were named following the HGVS nomenclature guidelines available at http://www.hgvs.org/mutnomen/. The NS-Euronet mutation database was used to check for mutation description at https://nseuronet.com/php/.

### PCR amplification method

We used an improved PCR protocol amplification which provides a robust detection of the recurrent intragenic deletion that removes exon4–10. This protocol has been previously described by Guevara et al. [28].

## Results

**Fig. 2:**
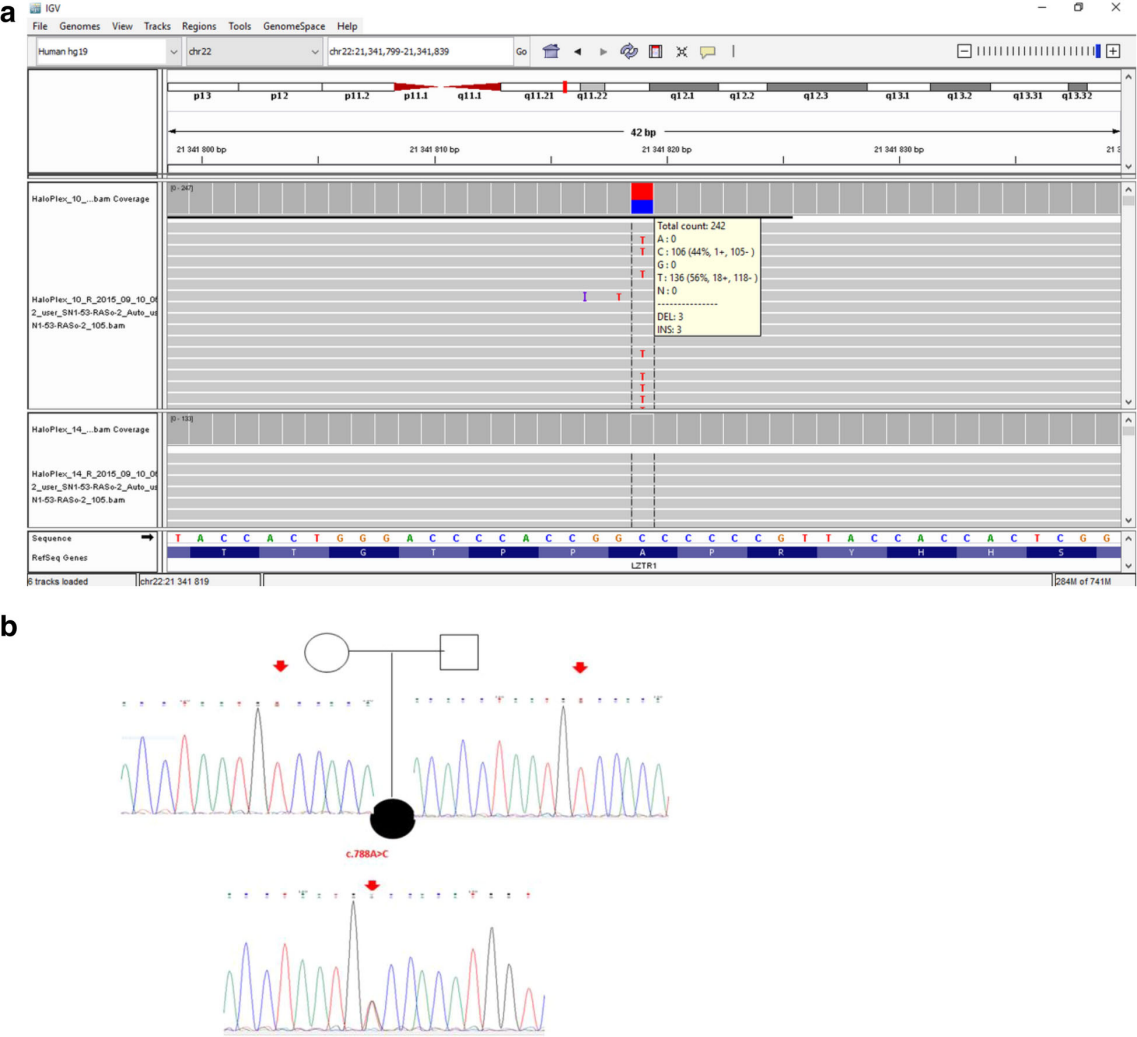
Sequencing results. **a** IGV browser visualization of the targeted NGS sequencing results showing the heterozygous c.788 T > G substitution in the RAF1 gene in the patient, (reverse sequence) which is absent in another NS patient (used as a control); **b** Sanger sequencing confirming the de novo appearance of the mutation in the patient, given its absence in the parents’ DNA samples

A total of 184 variants were detected across the 29 genes analyzed. Filtering these results using in silico software predictors of a mutation’s impact such as UMD predictor, Mutation-taster, PolyPhen and others, whose algorithms are integrated into the Varaft software, issued only one heterozygous variant as potentially pathogenic: a heterozygous SNV in exon 7 of the *RAF1* gene predicted to lead to a missense amino acid change (NM_002880: c.788 T > G, p. Val263Gly) (Fig. 2a). This was retained since it was correlated to the phenotypic description of the patient and was already reported [29].We confirmed this alteration in the patient by Sanger Sequencing. The analysis of transmission in the patients’ parents confirmed that it occurred as de novo mutation (Fig. 2b).

**Fig. 3:**
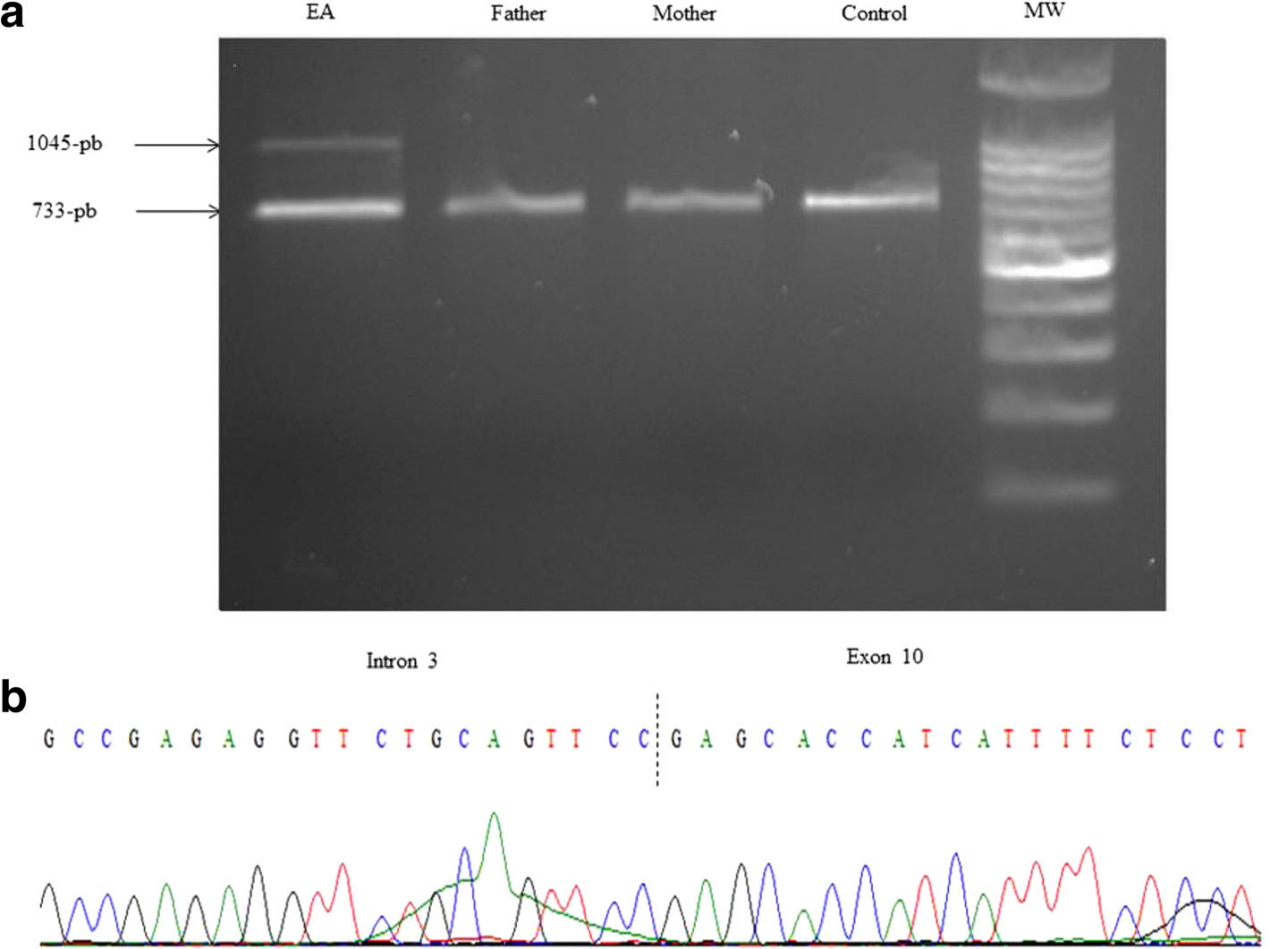
Molecular confirmation of IP. **a** Polymerase Chain Reaction (PCR) amplification of peripheral blood genomic DNA confirms the presence of the intragenic deletion in the *IKBKG* gene in the proband (the 1045 bp band) but not in her parents and in the control (the 733 bp band). **b** Sanger sequencing of the 1045 bp band from the proband’s amplified DNA confirms a breakpoint in intron 3

Presence of a de novo exon 4–10 deletion of *IKBKG* gene was demonstrated by PCR amplification in the proband (Fig. 3a) and was confirmed in the proband’s DNA using Sanger Sequencing of the 1045-bp band confirming a breakpoint in intron 3.(Fig. 3b).

## Discussion and conclusion

To the best of our knowledge, this is the first reported clinical case of NS associated with IP in a non-consanguineous female patient. As previously discussed, several causal genes are associated with the NS phenotype, including *PTPN11*, *SOS1, RAF1*, *BRAF*, *NRAS*, *SHOC2*, *CBL*, and *RIT1*.

Our patient harbored a mutation in *RAF1*, in which rare substitutions were previously reported in the context of NS and RASopathies [30–32]. RAF1 (OMIM, 164760) is a mitogen activated protein kinase kinase that constitutes, in addition to BRAF and ARAF*,* a small family of serine threonine kinases which relays signals from activated RAS proteins to the major effectors of this pathway, ERK1/2 via MEK1/2. Compared to BRAF, RAF1 and ARAF have considerably lower MEK kinase activity, different expression profiles and also distinct regulatory mechanisms: missense mutations in these two genes are thus rarely observed in malignancies [10]. The ubiquitously expressed *RAF1* encodes a 648 amino acids protein containing three functional and conserved domains: the N-terminal CR1 (amino acids residues 61–192), *CR2* (residues 251–266),, and CR3 kinase domain (residues 333–625) [33].

About 70% of all the *RAF1* mutations identified to date are amino acid substitutions located in the *CR2* domain. The second group of mutations, identified in 15% of NS or LS-causing *RAF1* mutations, affects the kinase domain and the last 15% of *RAF1* mutations affects two adjacent residues, located at the C-terminus. None of the mutations causing NS or LS are reported in a cancer context.

Our patient’s mutation is localized at position Val263, adjacent to the most frequently mutated residues, in the same CR2 domain. Functional characterizations support that mutations which affect the 14–3-3 binding motif (such is in our case) cause an enhanced kinase activity in downstream *RAS* signaling (*MEK* and *ERK*) [10, 29, 34]. The exact mechanism of *RAF1* activation in mutants remains unexplained.

Our patient showed a mutation that was previously described prenatally in a fetus showing evocative signs of Noonan syndrome (fetal hydrops and cystic hygromacolli) together with hypoplastic left heart syndrome (HLHS), one of the most severe congenital heart defects, characterized by underdevelopment of the structures in the left-aorta complex and detectable by ultrasound at prenatal sonography between 18 and 22 weeks of gestation of the fetal heart. In Noonan syndrome patients, HLHS has only been described twice [35]. The same mutation caused in our patient one of the two most frequent congenital heart defects observed in NS: pulmonic stenosis, suggesting that other factors (genetic or environmental) are involved in the determination of the cardiac anomaly, in addition to the *RAF1* p.Val263Gly mutation.

Gain of function mutations in *RAF1* were identified for the first time in 2007 in patients with NS and negative for *PTPN11*, *KRAS* and *SOS1* mutations and two of six patients with LEOPARD syndrome [31, 36], with a frequency ranging between 10 and 30%. Examination of detailed clinical manifestations of these patients showed a strong association among *RAF1* mutations and congenital cardiac defects (94% of children carrying *RAF1* gene mutation), namely hypertrophic cardiomyopathy (HCM) in 70–80% of the patients, which is significant compared to the 18% HCM prevalence observed in the general NS population [10], but atrial septal defects (28%), pulmonary valve stenosis (12%), and arrhythmia are also observed [37].

Craniofacial characteristics, skeletal abnormalities and mental retardation can also be present. Skin and hair anomaly are less marked, but multiple *naevi*, café-au-lait spots and lentigines were present in one-third of NS patients with *RAF1* mutations, that suggests a predisposition to hyperpigmented cutaneous lesions [10, 34, 38–40]. In IP affected females, major linear skin hyperpigmentation can be associated with a variety of ophthalmic disorders (strabismus, retinopathy, congenital cataract…), nail and teeth dysplasia and occasionally central nervous system disease ranging from seizures to severe motor and intellectual delay [21]. Differently to NS, skin lesions of IP occur in 4 classic cutaneous stages, observed in our patient: stage 1 is characterized by erythema, vesicles, and pustules; stage 2 by papules, verrucous lesions, and hyperkeratosis; stage 3 by hyperpigmentation; and stage 4 by pallor, atrophy, and scarring [41]. Nail dystrophy is frequent but usually mild [25].

The relatively low incidence of *RAF1* gene mutations in patients with NS and the much rarer onset of cutaneous disorders within this category of patients make it difficult to establish a clear genotype/phenotype correlation and to better explain the possible underlying mechanism of such a rare association of NS phenotypes. The association between NS due to a RAF1 mutation and another disease in one patient has been rarely reported. Cerebrovascular anomalies [42], Burkitt lymphoma [43] and usually cardiomyopathies [34, 44] have been associated to NS.

## Conclusion

The Co-occurence of two or several diseases has been recently reported in Tunisian population. This phenomenon has been observed mainly for autosomal recessive diseases and is due to the high rate of consanguinity [45, 46]. Nevertheless, the association we report between NS and IP is probably coincidental since no link has been established among the 2 pathophysiological pathways and high rates of consanguinity in the Tunisian patients’ population are not likely to influence the appearance of IP nor NS, whose inheritance patterns are not autosomal recessive.

## Abbreviations

CNV: Copy Number variation
HCM: Hypertrophic Cardiomyopathy
HLHS: Hypoplastic Left Heart Syndrome
IP: Incontinentia Pigmenti
NS: Noonan Syndrome
PCR: Polymeras Chain Reaction
RAS MAPK: Ras Mitogen Activated Protein Kinase

## References

[1] Roberts AE, Allanson JE, Tartaglia M, Gelb BD (2013). Noonan syndrome. Lancet.

[2] Turner AM. Noonan syndrome. J Paediatr Child Health. 2014;50:E14–E20. 10.1111/j.1440-1754.2010.01970.x 21771153

[3] Romano AA, Allanson JE, Dahlgren J, Gelb BD, Hall B, Pierpont ME (2010). Noonan syndrome: clinical features, diagnosis, and management guidelines. Pediatrics.

[4] Van der Burgt I, Berends E, Lommen E, Van Beersum S, Hamel B, Mariman E (1994). Clinical and molecular studies in a large Dutch family with Noonan syndrome. Am J Med Genet.

[5] Zenker M (2009). Genetic and pathogenetic aspects of Noonan syndrome and related disorders. Horm Res.

[6] Allanson JE, Bohring A, Dörr H-G, Dufke A, Gillessen-Kaesbach G, Horn D (2010). The face of Noonan syndrome: does phenotype predict genotype. Am J Med Genet A.

[7] Bhambhani V, Muenke M (2014). Noonan syndrome. Am Fam Physician.

[8] AAL J, Malaquias AC, IJP A, Mendonca BB (2009). Noonan syndrome and related disorders: a review of clinical features and mutations in genes of the RAS/MAPK pathway. Horm Res.

[9] Narumi Y, Aoki Y, Niihori T, Sakurai M, Cavé H, Verloes A (2008). Clinical manifestations in patients with SOS1 mutations range from Noonan syndrome to CFC syndrome. J Hum Genet.

[10] Tartaglia M, Zampino G, Gelb BD (2010). Noonan syndrome: clinical aspects and molecular pathogenesis. Mol Syndromol.

[11] Nshuti S, Hategekimana C, Uwineza A, Hitayezu J, Mucumbitsi J, Rusingiza EK, et al. Patients with Noonan Syndrome phenotype : spectrum of clinical features and congenital heart defect. Rwanda Medical Journal 2010;68:26–312010;68:26–31.

[12] Tumurkhuu M, Saitoh M, Sato A, Takahashi K, Mimaki M, Takita J (2010). Comprehensive genetic analysis of overlapping syndromes of RAS/RAF/MEK/ERK pathway. Pediatr Int.

[13] Gelb BD, Tartaglia M (2006). Noonan syndrome and related disorders: dysregulated RAS-mitogen activated protein kinase signal transduction. Hum Mol Genet.

[14] Tidyman WE, Rauen KA (2009). The RASopathies: developmental syndromes of Ras/MAPK pathway dysregulation. Curr Opin Genet Dev.

[15] Tartaglia M, Kalidas K, Shaw A, Song X, Musat DL, van der Burgt I (2002). PTPN11 mutations in Noonan syndrome: molecular spectrum, genotype-phenotype correlation, and phenotypic heterogeneity. Am J Hum Genet.

[16] Tartaglia M, Martinelli S, Stella L, Bocchinfuso G, Flex E, Cordeddu V (2006). Diversity and functional consequences of germline and somatic PTPN11 mutations in human disease. Am J Hum Genet.

[17] Aoki Y, Niihori T, Narumi Y, Kure S, Matsubara Y (2008). The RAS/MAPK syndromes: novel roles of the RAS pathway in human genetic disorders. Hum Mutat.

[18] Tartaglia M, Gelb BD, Zenker M (2011). Noonan syndrome and clinically related disorders. Best Pract Res Clin Endocrinol Metab.

[19] Rauen KA, Huson SM, Burkitt-Wright E, Evans DG, Farschtschi S, Ferner RE (2015). Recent developments in neurofibromatoses and RASopathies: management, diagnosis and current and future therapeutic avenues. Am J Med Genet Part A.

[20] Bardaro T, Falco G, Sparago A, Mercadante V, Molins EG, Tarantino E (2003). Two cases of misinterpretation of molecular results in incontinentia pigmenti, and a PCR-based method to discriminate NEMO/IKKγ gene deletion. Hum Mutat.

[21] Fusco F, Paciolla M, Conte MI, Pescatore A, Esposito E, Mirabelli P (2014). Incontinentia pigmenti: report on data from 2000 to 2013. Orphanet J Rare Dis.

[22] Ormerod AD, White MI, McKay E, Johnston AW (1987). Incontinentia pigmenti in a boy with Klinefelter’s syndrome. J Med Genet.

[23] Fusco F, Bardaro T, Fimiani G, Mercadante V, Miano MG, Falco G (2004). Molecular analysis of the genetic defect in a large cohort of IP patients and identification of novel NEMO mutations interfering with NF-κB activation. Hum Mol Genet.

[24] Conte MI, Pescatore A, Paciolla M, Esposito E, Miano MG, Lioi MB (2014). Insight into IKBKG/NEMO locus: report of new mutations and complex genomic rearrangements leading to incontinentia pigmenti disease. Hum Mutat.

[25] Minić S, Trpinac D, Obradović M (2014). Incontinentia pigmenti diagnostic criteria update. Clin Genet.

[26] Wang K, Li M, Hakonarson H (2010). Nucleic Acids Res.

[27] Salgado D, Desvignes JP, Rai G, Blanchard A, Miltgen M, Pinard A (2016). UMD-predictor: a high-throughput sequencing compliant system for pathogenicity prediction of any human cDNA substitution. Hum Mutat.

[28] Guevara BEK, Hsu CK, Liu L, Feast A, Alabado KLP, Lacuesta MPM (2016). Improved molecular diagnosis of the common recurrent intragenic deletion mutation in IKBKG in a Filipino family with incontinentia pigmenti. Aust J Dermatol.

[29] Schulz S, Fröber R, Kraus C, Schneider U (2012). Prenatal diagnosis of hypoplastic left heart syndrome associated with Noonan syndrome and de novo RAF1 mutation. Prenat Diagn.

[30] Ko JM, Kim JM, Kim GH, Yoo HW (2008). PTPN11, SOS1, KRAS, and RAF1 gene analysis, and genotype-phenotype correlation in Korean patients with Noonan syndrome. J Hum Genet.

[31] Razzaque MA, Nishizawa T, Komoike Y, Yagi H, Furutani M, Amo R (2007). Germline gain-of-function mutations in RAF1 cause Noonan syndrome. Nat Genet.

[32] Kuburović V, Vukomanović V, Carcavilla A, Ezquieta-Zubicaray B, Kuburović N (2011). Two cases of LEOPARD syndrome - RAF1 mutations firstly described in children. Turk J Pediatr.

[33] Wellbrock C, Karasarides M, Marais R (2004). The RAF proteins take Centre stage. Nat Rev Mol Cell Biol.

[34] Sana ME, Spitaleri A, Spiliotopoulos D, Pezzoli L, Preda L, Musco G (2014). Identification of a novel de novo deletion in RAF1 associated with biventricular hypertrophy in Noonan syndrome. Am J Med Genet Part A.

[35] Antonelli D, Antonelli J, Rosenfeld T (1990). Noonan’s syndrome associated with hypoplastic left heart. Cardiology.

[36] Kobayashi T, Aoki Y, Niihori T, Cavé H, Verloes A, Okamoto N (2010). Molecular and clinical analysis of RAF1 in Noonan syndrome and related disorders: dephosphorylation of serine 259 as the essential mechanism for mutant activation. Hum Mutat.

[37] Pandit B, Sarkozy A, Pennacchio LA, Carta C, Oishi K, Martinelli S (2007). Gain-of-function RAF1 mutations cause Noonan and LEOPARD syndromes with hypertrophic cardiomyopathy. Nat Genet.

[38] Kneitel AW, Norby A, Vettraino I, Treadwell MC (2015). A novel mutation on *RAF1* in association with fetal findings suggestive of Noonan syndrome. Fetal Pediatr Pathol.

[39] Wu X, Simpson J, Hong JH, Kim K, Thavarajah NK, Backx PH (2011). MEK-ERK pathway modulation ameliorates disease phenotypes in a mouse model of Noonan syndrome associated with the Raf1 L613V mutation. Differences.

[40] Tartaglia M, Gelb BD (2010). Disorders of dysregulated signal traffic through the RAS-MAPK pathway: phenotypic spectrum and molecular mechanisms. Ann N Y Acad Sci.

[41] Young I, Wilkie A (1994). Syndrome of the month. J Med Genet.

[42] Zarate YA, Lichty AW, Champion KJ, Clarkson LK, Holden KR, Matheus MG (2013). Unique cerebrovascular anomalies in Noonan syndrome with RAF1 mutation. J Child Neurol.

[43] Cianci P, Tono V, Sala A, Locatelli L, Carta C, Rizzari C (2013). A boy with Burkitt lymphoma associated with Noonan syndrome due to a mutation in RAF1. Am J Med Genet A.

[44] Gelb BD, Roberts AE, Tartaglia M (2015). Cardiomyopathies in Noonan syndrome and the other RASopathies. Prog Pediatr Cardiol.

[45] Ben Abdallah LC, Lakhoua Y, Nagara M, Khiari K, Elouej S, Messaoud O (2014). A tunisian patient with two rare syndromes: triple a syndrome and congenital hypogonadotropic hypogonadism. Horm Res Paediatr.

[46] Romdhane L, Messaoud O, Bouyacoub Y, Kerkeni E, Naouali C, Cherif Ben Abdallah L (2016). Comorbidity in the Tunisian population. Clin Genet.

